# Integrative multi-omics analysis of gastric cancer evolution from precancerous lesions to metastasis identifies a deep learning-based prognostic model

**DOI:** 10.3389/fimmu.2025.1680517

**Published:** 2025-10-31

**Authors:** Yulin Ren, Xiaoyan Zhang, Ke Li, Shuning Xu, Lei Qiao, Qun Li, Cheng Zhang, Ying Liu

**Affiliations:** ^1^ Department of Medical Oncology, The Affiliated Cancer Hospital of Zhengzhou University and Henan Cancer Hospital, Zhengzhou, China; ^2^ State Key Laboratory of Experimental Hematology and Division of Pediatric Blood Diseases Center, Institute of Hematology and Blood Diseases Hospital, Peking Union Medical College, Chinese Academy of Medical Sciences, Tianjin, China

**Keywords:** gastric cancer, single-cell RNA sequencing, tumor microenvironment, WGCNA, deep learning prognostic model

## Abstract

**Background:**

Gastric cancer progression involves complex interactions among tumor cells, immune components, and stromal elements within the tumor microenvironment. However, a comprehensive understanding of cellular heterogeneity, spatial organization, and cell-cell communication in gastric cancer remains incomplete.

**Methods:**

Single-cell RNA sequencing was performed on 252, 399 cells from six tissue types, spanning gastritis, intestinal metaplasia, primary tumors, adjacent normal tissue, and metastatic lesions. Integration with spatial transcriptomics enabled spatial mapping of cellular interactions. Pseudotime, cell-cell communication, and transcriptional heterogeneity analyses were conducted. Tumor stage-associated gene modules were identified using Weighted Gene Co-expression Network Analysis (WGCNA) of The Cancer Genome Atlas (TCGA) data. Finally, a deep learning-based prognostic model was developed and externally validated.

**Results:**

Our analysis revealed dynamic remodeling of the tumor microenvironment during gastric cancer progression, characterized by the expansion of dysfunctional CD8+ T cells, pro-tumorigenic fibroblasts (e.g., ITGBL1+, PI16+, and ITLN1+), and altered myeloid populations. Stromal-immune crosstalk, particularly fibroblast-driven immunosuppressive signaling, was prominent. Spatial transcriptomics revealed the colocalization of immune and stromal cells, supporting spatially organized cellular interactions. WGCNA identified a gene module (657 genes) associated with T cell, myeloid, and stromal alterations, as well as tumor stage. A deep learning model based on this gene set accurately stratified patients according to survival in both TCGA and independent validation cohorts. Risk scores were correlated with clinical features, including tumor stage and therapeutic response.

**Conclusions:**

Our integrative single-cell, spatial, and computational analysis provides a high-resolution map of gastric cancer microenvironment remodeling. We identified key stromal and immune subpopulations, extensive cellular communication networks, and spatial structures that collectively drive tumor progression and metastasis. The derived gene signature and prognostic model have the potential for clinical risk stratification and therapeutic targeting in gastric cancer.

## 1 Introduction

Gastric cancer (GC) remains a major global health burden, ranking fifth as the most common malignancy and the fourth leading cause of cancer-related mortality worldwide (1). Despite significant progress in diagnosis and treatment, the prognosis of GC, particularly at advanced stages, remains dismal largely because of late detection, tumor heterogeneity, and metastasis (2, 3). Increasing evidence indicates that tumor progression is not solely determined by malignant epithelial cells but is intricately regulated by the surrounding tumor microenvironment (TME), which consists of diverse immune and stromal cell populations (4, 5).

The GC microenvironment is characterized by substantial cellular heterogeneity and dynamic interactions between tumor cells, fibroblasts, endothelial cells (ECs), and immune components. For example, cancer-associated fibroblasts have been shown to promote extracellular matrix (ECM) remodeling, immunosuppression, and metastatic dissemination (6, 7). Similarly, dysfunction and exhaustion of tumor-infiltrating CD8+ T cells and accumulation of immunosuppressive myeloid cells have been associated with poor clinical outcomes (8). However, most conventional transcriptomic studies based on bulk tissue analysis lack the resolution required to capture the spatial and cellular complexities of the TME.

Recent advances in single-cell RNA sequencing (scRNA-seq) and spatial transcriptomics have enabled the unprecedented characterization of the cellular landscape and spatial organization of solid tumors, including GC (9–11). These technologies have revealed key immune-stromal interactions, novel cell subpopulations, and cellular programs driving tumor progression (12, 13). Nevertheless, comprehensive multidimensional analyses integrating single-cell, spatial, and clinical data to elucidate the progression and prognosis of GC remain limited.

Simultaneously, deep learning approaches have emerged as powerful tools for biomarker discovery and outcome prediction in oncology. Deep neural networks, which capture complex nonlinear patterns in high-dimensional data, have demonstrated superior performance to traditional statistical models in tasks such as patient risk stratification and survival prediction across multiple cancer types (14, 15). Notably, deep learning-based prognostic models that leverage transcriptomic or histopathological features have shown promise in GC; however, few studies have incorporated biologically grounded features derived from single-cell and spatial analyses (16, 17).

In this study, we present an integrative framework that combines scRNA-seq, spatial transcriptomics, bulk transcriptomic profiling, and deep learning to dissect the cellular ecosystem and molecular underpinnings of GC progression. We systematically mapped the dynamic remodeling of the immune and stromal compartments, characterized intercellular communication networks, and identified gene modules associated with tumor stage and microenvironmental alterations. Furthermore, we developed and validated a deep learning-based prognostic model based on single-cell-derived biological signatures, offering new insights into the mechanisms driving GC progression and providing clinically relevant tools for patient risk stratification.

## 2 Materials and methods

### 2.1 Human samples and ethical approval

A total of 77 tissue samples representing different pathological states of GC progression were collected from public databases, including non-atrophic gastritis (n = 3), chronic atrophic gastritis (n = 3), intestinal metaplasia (IM, n = 6), adjacent normal gastric tissues (n = 14), primary GC (n = 36), and distant metastases (n = 15). All samples were obtained from the Gene Expression Omnibus (GEO) database (GSE134520, GSE183904, GSE206785, GSE163558, and GSE234129) (https://www.ncbi.nlm.nih.gov/geo/). The training set data of the deep learning prognosis model were obtained from The Cancer Genome Atlas (TCGA) database (TCGA-STAD), and the validation set data were obtained from the GEO database (GSE84433). Studies involving human participants were reviewed and approved by the Medical Ethics Committee of Henan Cancer Hospital. Written informed consent was obtained from all participants or their relatives.

### 2.2 scRNA-seq and data preprocessing

scRNA-seq data were processed and analyzed using the Scanpy package for Python (version 1.9.0) (18). Scrublet was used to identify potential doublets in each sample, and, after that, cells with scrublet score > 0.5 were filtered out as doublets. Subsequent filtering was performed to exclude empty droplets and doublet cells based on the following criteria: cells were retained only if they contained between 500 and 5, 000 detected genes and 700 to 50, 000 UMI counts. Cells with more than 10% mitochondrial genes expressed were removed as potential low-quality cells.

Initial gene count were first normalized for library size using sc.pp.normalize_total(adata, target_sum=1e4) to 10, 000 counts per cell, making expression levels comparable across cells. Normalized data were then log-transformed with sc.pp.log1p(adata). Highly variable genes (HVGs) were identified using sc.pp.highly_variable_genes(adata, layer=“counts”, n_top_genes=2000). To remove unwanted variation associated with sequencing depth, total counts per cell were regressed out using sc.pp.regress_out (adata, [“total_counts”], layer=“scaled”), followed by scaling of the data to unit variance with a maximum value of 10 [sc.pp.scale(adata, max_value=10, layer=“scaled”)]. Downstream dimensionality reduction and clustering analyses were then performed on the processed data.

The BBKNN algorithm was applied for dimensionality reduction and batch effect correction (19). In this framework, continuous technical variables, such as mitochondrial gene content, were modeled as covariates, whereas donor identity was treated as a categorical batch factor. The neighborhood graph was constructed using scanpy.pp.neighbors with k set to 30, followed by community detection using the Leiden algorithm (scanpy.tl.leiden), with a resolution of 1.

Differentially expressed genes (DEGs) across clusters were identified using scanpy.tl.rank_genes_groups, with statistical significance determined by a t-test combined with Benjamini-Hochberg false discovery rate correction. Only DEGs meeting the following criteria were retained for downstream analyses: log_2_ fold change > 1, expression detected in at least 10% of the cells within the cluster, and Bayes factor > 2.

### 2.3 Dimensionality reduction, clustering, and cell type annotation

Principal component analysis was performed on the highly variable genes, followed by Uniform Manifold Approximation and Projection (UMAP) for dimensionality reduction. Unsupervised clustering was performed by using the Leiden algorithm implemented in Scanpy. Cell types and subtypes were annotated based on the expression of canonical marker genes.

### 2.4 Differential abundance and transcriptional heterogeneity analysis

The proportion of each cell type across the tissue types was calculated and compared using the Wilcoxon rank-sum test. Jensen-Shannon divergence was used to quantify transcriptional heterogeneity within major cell lineages, following established methods (20).

### 2.5 Trajectory inference and pseudotime analysis

To infer the cellular differentiation trajectories, Monocle 2 (version 2.18.0) was applied to NK/T cells using highly variable genes for dimensionality reduction and pseudotime ordering (21).

### 2.6 Intercellular communication analysis

Cell-cell interactions were inferred using CellChat (version 1.1.3) (22). Interaction probabilities were computed based on known ligand-receptor pairs, and global and pathway-specific communication networks were constructed. The strengths of the outgoing and incoming interactions were quantified for each cell subtype.

### 2.7 Spatial mapping of cell types using cell2location

To map single-cell transcriptomic profiles onto spatial transcriptomic data and infer the spatial distribution of distinct cell types, we applied the probabilistic model cell2location (23). This approach enables robust deconvolution of spatial transcriptomic data by leveraging reference cell-type signatures derived from single-cell RNA sequencing. Cell type-specific gene expression signatures were obtained from an annotated scRNA-seq dataset. These signatures were then used as prior information in cell2location to estimate the spatial abundance of each cell type across tissue sections generated by the 10x Genomics Visium platform. The model was trained using default hyperparameters as recommended by the developers. The resulting spatial abundance maps provided high-resolution predictions of cell-type localization within the tissue architecture. These maps were visualized using Scanpy frameworks, confirming the colocalization of immune and stromal cells within the tumor regions. This spatial distribution supported the predicted intercellular interactions inferred from cell-cell communication analyses and revealed the organizational basis for immune-stromal crosstalk within the TME.

### 2.8 ssGSEA and WGCNA

Gene expression and clinical data of patients with GC were downloaded from TCGA database. Single-sample Gene Set Enrichment Analysis (ssGSEA) was used to quantify transcriptional alterations in T cells, macrophages, and stromal cells across the samples (24). Weighted Gene Co-expression Network Analysis (WGCNA) (version 1.71) was used to identify the gene modules associated with immune alterations and tumor stages (25). Module-trait correlations were assessed, and significant modules were selected for further prognostic model construction.

### 2.9 Deep learning-based prognostic model construction and validation

To construct a robust prognostic model for GC, we implemented DeepSurv, a deep learning extension of the traditional Cox proportional hazards model specifically designed to capture complex nonlinear relationships between input features and survival risk (26). The gene expression profiles of the 657 stage-associated module genes identified via WGCNA were used as input features for model training.

The model architecture consisted of fully connected feedforward layers with ReLU activation functions. Batch normalization and dropout regularization were applied to prevent overfitting. The network was optimized using the Adam optimizer, with hyperparameters, including learning rate, dropout rate, and hidden layer dimensions, tuned via fivefold cross-validation within the training cohort (TCGA-STAD dataset).

The model output was a continuous risk score that reflected the predicted hazard function for each patient. The patients were stratified into high- and low-risk groups based on the median risk score. Kaplan-Meier survival analysis and log-rank tests were performed to assess survival differences between the risk groups. Model performance was further evaluated using time-dependent receiver operating characteristic (ROC) curves, with area under the curve values calculated at 1, 3, and 5 years.

To assess model generalizability, the trained DeepSurv model was applied to an independent external validation cohort (GSE84433). Risk scores were computed for each patient, and the performance of survival prediction was evaluated using the same procedures as in the training set. Additionally, the association between the DeepSurv-derived risk scores and clinicopathological features, including tumor stage, lymph node status, and treatment information, was explored using appropriate statistical tests.

### 2.10 Flow cytometry of tumor-infiltrating T cells

Primary tumor tissue, adjacent normal tissue, and ascitic fluid from peritoneal metastasis were obtained from a patient with GC. The samples were processed within 2 hours of collection.

Solid tissues were cut into small fragments and digested in RPMI-1640 containing 1 mg/mL collagenase IV and 0.1 mg/mL DNase I at 37 °C for 30 min. The cell suspensions were filtered through a 70 μm strainer. Red blood cells were removed using the ACK lysis buffer. The ascitic cells were collected by centrifugation and washed with PBS. Cells were stained with a fixable viability dye (Invitrogen), followed by incubation with antibodies against CD45, CD3, CD4, CD8, and PD-1 (BioLegend). For intracellular FOXP3 staining, cells were fixed and permeabilized using a transcription factor buffer set (eBioscience) and incubated with an anti-FOXP3 antibody. Flow cytometry was performed using the BD LSRFortessa. Data were analyzed using FlowJo software (v10). CD8^+^ Tex cells were defined as CD45^+^CD3^+^CD8^+^PD-1^+^. Regulatory T cells (Tregs) were defined as CD45^+^CD3^+^CD4^+^FOXP3^+^. The frequencies were calculated as the percentage of live CD45^+^ cells.

### 2.11 RNA extraction and quantitative PCR

Total RNA was extracted from adjacent non-tumor tissue, primary gastric tumor tissue, and peritoneal metastases obtained from patients with GC using a Universal RNA Purification Kit (EZBioscience, EZB-RN4), following the manufacturer’s instructions. Subsequently, 400 ng of total RNA was reverse-transcribed into cDNA using the PrimeScript RT Master Mix Kit (TaKaRa, RR036A). For quantitative PCR analysis, 100 ng of cDNA was used per reaction with PerfectStart Green qPCR SuperMix (TransGen, AQ601-04) in a real-time PCR system (Roche, LightCycler 480). Gene expression levels were normalized to TATA-binding protein as the internal control.

### 2.12 Statistical analysis

Statistical analyses were performed in R (version 4.1.2) unless otherwise specified. For comparisons between groups, Wilcoxon rank-sum tests or Kruskal-Wallis tests were applied, as appropriate. Differences in survival were assessed using the log-rank test. Statistical significance was set at P < 0.05.

## 3 Results

### 3.1 Construction of a comprehensive single-cell transcriptomic atlas across gastric disease states and metastatic sites

**Figure 1:**
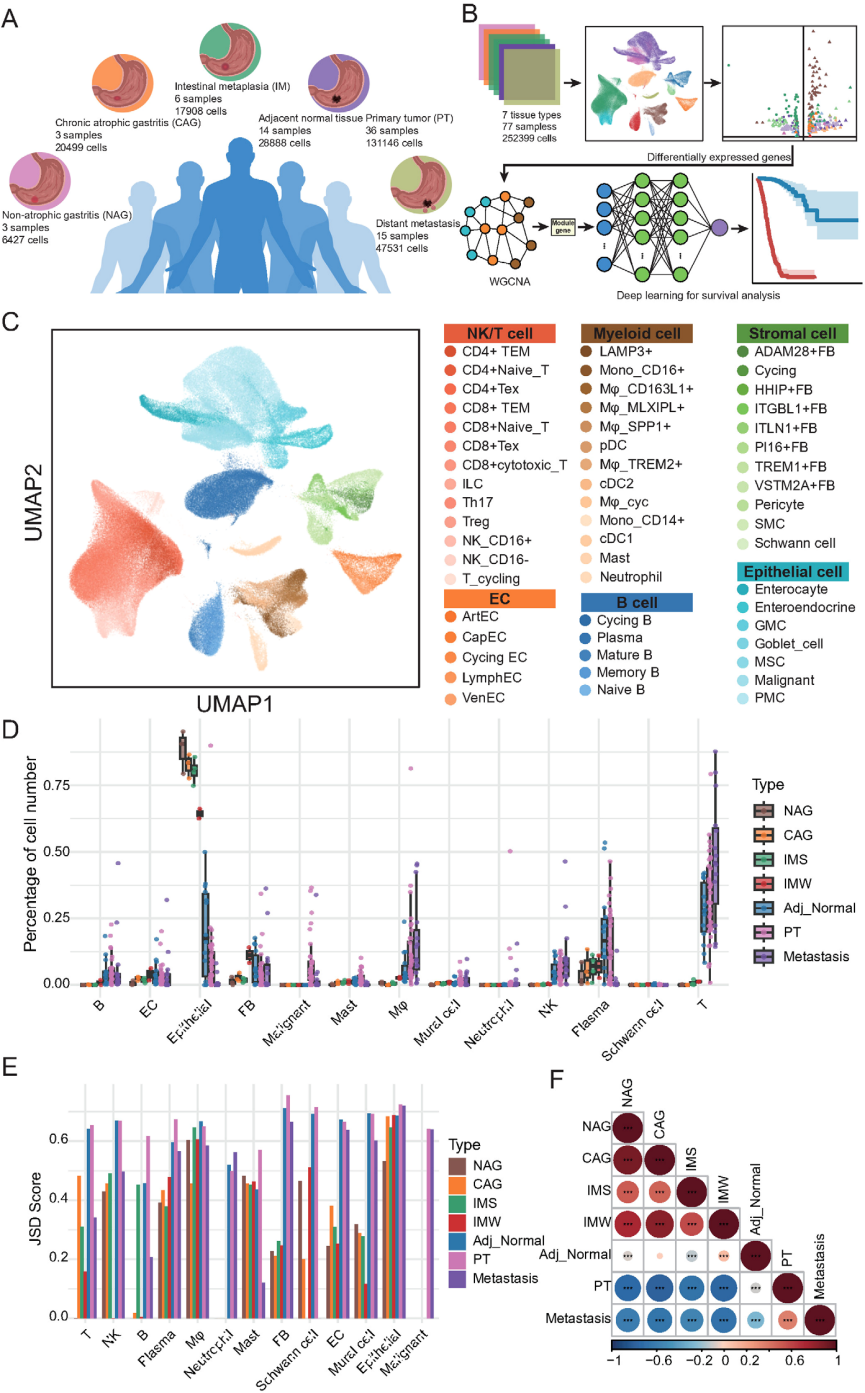
Single-cell transcriptomic atlas reveals cellular heterogeneity and tissue-specific remodeling across gastric disease states and metastases. **(A)** Schematic overview of tissue types and sample distribution included in this study. Single-cell RNA sequencing (scRNA-seq) was performed on 77 samples from 7 tissue types: non-atrophic gastritis (NAG, n = 3), chronic atrophic gastritis (CAG, n = 3), intestinal metaplasia (wild-type IM [IMW; n = 3] and severe IM [IMS; n = 3]), adjacent normal tissues (n = 14), primary tumors (PT, n = 36), and distant metastases (n = 15). **(B)** Workflow of scRNA-seq data analysis and downstream approaches. Data from 252, 399 cells were integrated for clustering and annotation. Differentially expressed genes were identified across stage types and subsequently subjected to WGCNA to detect gene modules associated with key cellular populations. These modules were incorporated into a deep learning model for survival prediction. **(C)** UMAP visualization of single cells colored by major lineages and annotated subtypes, including epithelial, stromal, endothelial, myeloid, B, T/NK, and malignant cells. **(D)** Boxplots showing the relative abundance of major cell types across the seven tissue types. Each dot represents one biological sample. **(E)** Bar plot of Jensen-Shannon Divergence (JSD) scores depicting intra-lineage transcriptional heterogeneity of major cell types across tissue types. **(F)** Heatmap of Spearman correlation coefficients reflecting global cellular composition similarities among the seven tissue types. Color scale represents correlation strength; asterisks indicate statistical significance (*p < 0.05, **p < 0.01, ***p < 0.001).

To systematically dissect cellular heterogeneity and microenvironmental alterations associated with GC initiation, progression, and metastasis, we performed scRNA-seq on 77 tissue samples from seven distinct pathological conditions, yielding 252, 399 high-quality cells (**Figures 1A, B**). These included samples from non-atrophic gastritis (n = 3, 6, 427 cells), chronic atrophic gastritis (n = 3, 20, 499 cells), IM (n = 6, 17, 908 cells, including 3 wild-type IM and 3 severe IM), adjacent normal gastric tissues (n = 14, 28, 888 cells), primary gastric tumors (n = 36, 131, 146 cells), and distant metastatic lesions (n = 15, 47, 531 cells). Unbiased clustering and dimensionality reduction were applied to delineate the cellular composition across these tissue types (**Figure 1C**; **Supplementary Figures 1A, B**). Based on canonical marker genes, we identified major cell lineages, including epithelial cells, stromal cells, ECs, myeloid cells, B cells, T/NK cells, and their corresponding subpopulations, thereby providing a comprehensive cellular map of gastric disease states.

Next, we quantitatively compared the relative abundance of each major cell lineage across seven tissue types (**Figure 1D**; **Supplementary Table 1**). Notably, macrophages exhibited a marked increase in cell proportion in cancer-adjacent tissues, primary tumors, and metastatic sites compared with gastritis and IM, suggesting their potential involvement in tumor-promoting processes. Similarly, T cells displayed a progressive increase in proportion from adjacent normal tissues to primary tumors and metastatic sites, whereas their abundance remained relatively low in gastritis samples. The NK cells showed comparable enrichment patterns. B cells were substantially enriched in adjacent normal, primary tumor, and metastatic tissues, with plasma cells exhibiting increased abundance in adjacent and primary tumor tissues but reduced representation in metastases. Mast cells showed a slight increase, specifically within primary tumor tissues, whereas neutrophils remained consistently scarce across all tissue types. Within the stromal compartment, EC proportions peaked during the gastritis and precancerous stages, but declined sharply in primary tumors and metastases. Fibroblasts were most abundant in IM, followed by a gradual decrease in cancer-adjacent primary tumors and metastatic tissues. Mural cells mirrored the trends observed in the fibroblasts. As expected, epithelial cells were predominant in precancerous lesions but were significantly reduced in tumor tissues, whereas malignant epithelial cells specifically emerged in primary tumor and metastatic samples.

To further investigate the transcriptional heterogeneity within each major cell lineage, we computed the Jensen-Shannon divergence across tissue types (**Figure 1E**; **Supplementary Table 2**). T cells exhibited reduced heterogeneity during gastritis and precancerous stages, followed by a sharp increase in primary tumor and metastatic tissues, suggesting transcriptional diversification during malignant transformation and dissemination. NK and plasma cells displayed progressive increases in heterogeneity from precancerous to tumor tissues. In contrast, fibroblasts, ECs, and mural cells showed low heterogeneity in gastritis and precancerous stages, with a marked increase in tumor and metastatic tissues, indicating that TME remodeling is associated with disease progression. Finally, correlation analysis of the global cellular composition revealed strong similarities between gastritis and precancerous tissues, whereas primary tumors and metastatic lesions displayed distinct cellular profiles and were negatively correlated with non-malignant tissues (**Figure 1F**; **Supplementary Table 3**). Adjacent normal tissues exhibited intermediate cellular features that partially resembled those of precancerous and tumor tissues. Collectively, these results delineate a stage-independent, disease-specific cellular remodeling process during GC development and metastasis, highlighting substantial alterations in the immune and stromal compartments.

### 3.2 Dynamic remodeling and functional heterogeneity of NK and T cell subsets during GC progression and metastasis

**Figure 2:**
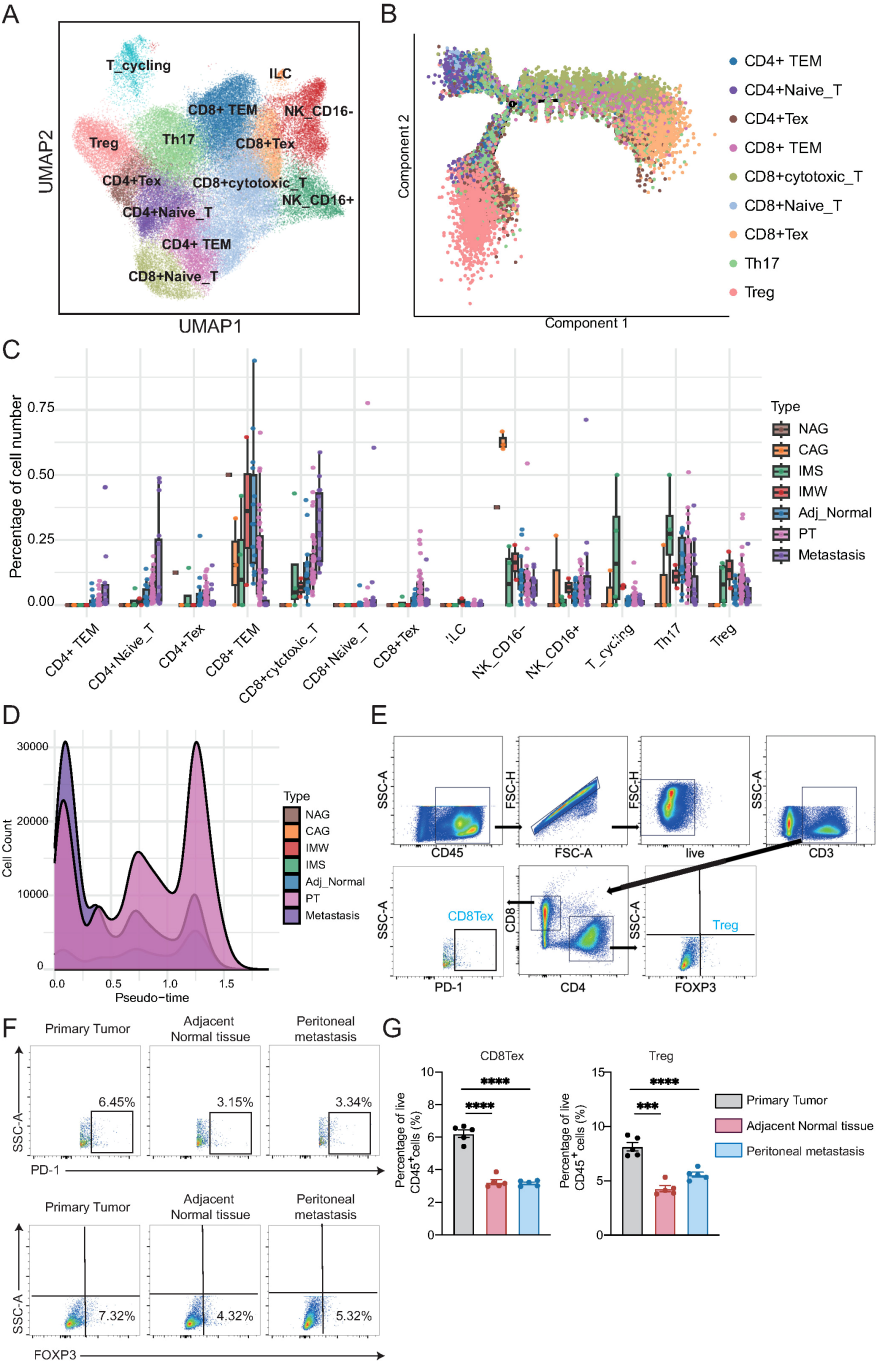
Transcriptional heterogeneity and trajectory dynamics alterations of NK and T cells during gastric cancer progression. **(A)** UMAP plot showing 13 transcriptionally distinct NK/T cell subpopulations, including CD4+ and CD8+ T cell subsets, innate lymphoid cells (ILC), and NK cell subsets. **(B)** Pseudotime trajectory of T cells inferred by Monocle. Naïve T cells reside at the trajectory root, with bifurcation into CD4+ and CD8+ differentiation branches. **(C)** Boxplots showing the relative abundance of NK/T cell subtypes across tissue types. Each dot represents one biological sample. CD4+ TEM, CD4+ naïve T, and CD8+ cytotoxic T cells increase from adjacent tissue to tumors and metastases, whereas CD8+ TEM, Th17, and NK_CD16− cells decline. **(D)** Density plot of pseudotime distribution for T cells from each tissue type, highlighting enrichment of metastatic cells at early differentiation states and broad distribution of primary tumor-derived cells. **(E)** Gating strategy for identifying CD8^+^PD-1^+^ (CD8^+^ Tex) and CD4^+^FOXP3^+^ (Treg) cells from live CD45^+^CD3^+^ T cells. **(F)** Representative flow plots from one patient. CD8^+^ Tex and Treg cells were more abundant in the primary tumor than in adjacent normal tissue or lymph node metastasis. **(G)** Statistical analysis of CD8^+^ Tex and Treg proportions from gastric cancer patients (n = 5/group). One-way ANOVA; ***P < 0.001, ****P < 0.0001.

To dissect the dynamic changes and functional heterogeneity of NK and T cells during GC development and metastasis, we performed detailed subclustering and trajectory inference analyses of NK/T cells derived from all tissue types. Unsupervised clustering revealed 13 distinct NK/T cell subpopulations, including CD4+ effector memory T cells (CD4+ TEM), CD4+ naïve T cells, CD4+ exhausted T cells (CD4+ Tex), CD8+ TEM, CD8+ cytotoxic T cells, CD8+ naïve T cells, CD8+ Tex, innate lymphoid cells, NK_CD16−, NK_CD16+, cycling T cells, Th17 cells, and Tregs (**Figure 2A**). Each cell subpopulation exhibited unique signature genes (**Supplementary Figure 2A**). Notably, the CD4+ and CD8+ T cell compartments exhibited transcriptionally distinct subclusters, reflecting functional diversification. To elucidate the potential differentiation trajectories of T cells, we performed a pseudotime analysis using Monocle (**Figure 2B**). Naïve CD4+ and Naïve CD8+ T cells resided at the root of the trajectory, consistent with their early differentiation state (**Supplementary Figure 2B**; **Supplementary Table 4**). The trajectory bifurcated into two major branches: one leading toward CD4+ effector (CD4+ TEM, CD4+ Tex, Th17, Treg) cells and the other toward CD8+ effector (CD8+ cytotoxic T, CD8+ TEM, CD8+ Tex) cells, reflecting lineage-specific differentiation and functional maturation (**Supplementary Figure 2C**).

Next, we quantified the proportions of NK/T cell subtypes across different tissue types (**Figure 2C**; **Supplementary Table 5**). CD4+ TEM, CD4+ naïve T cells, and CD8+ cytotoxic T cells exhibited progressive enrichment from cancer-adjacent to primary tumors and metastatic tissues, suggesting their potential involvement in antitumor responses. In contrast, CD8+ TEM, Th17 cells, and NK_CD16− cells displayed a marked decline in abundance along disease progression. Furthermore, we examined the distribution of T cells along the pseudotime trajectory across different tissue types (**Figure 2D**; **Supplementary Table 4**). Cells from metastatic lesions were enriched in early pseudotime states, whereas primary tumor samples displayed a broad distribution along the trajectory, implying distinct differentiation dynamics between the primary and metastatic sites. To further validate these findings, we collected matched samples, including the primary tumor, cancer-adjacent tissue, and ascitic fluid from peritoneal metastases, from the same patient with GC, and performed flow cytometry. Consistent with the single-cell data, we observed a significantly higher proportion of CD8^+^ Tex and Treg cells in the primary tumor than in the adjacent normal tissue and peritoneal metastasis (**Figures 2E–G**). These results suggest that the immunosuppressive microenvironment, characterized by the presence of exhausted and regulatory T cells, is more prominent in primary lesions.

**Figure 3:**
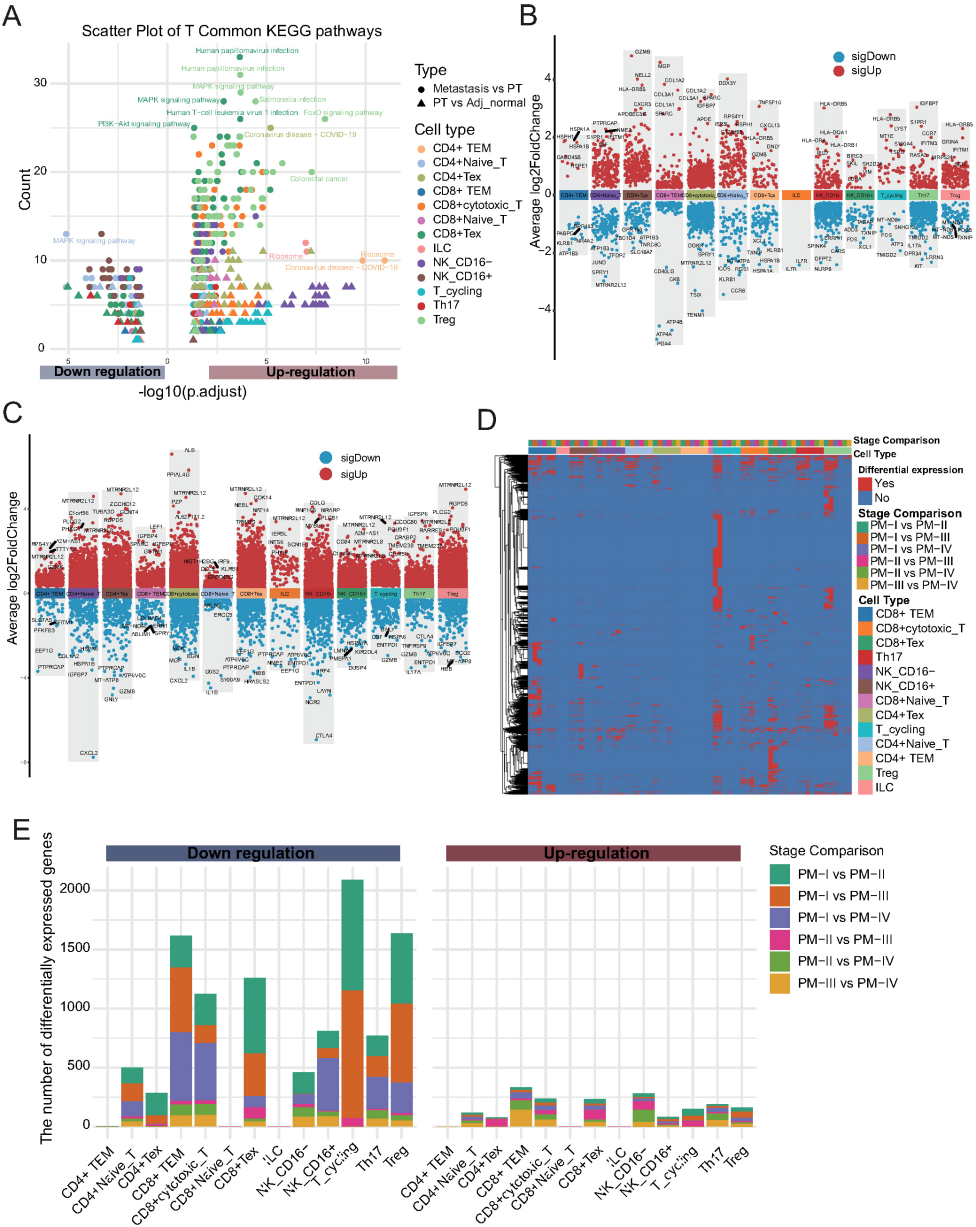
Stage-dependent alterations of NK and T cells during gastric cancer progression. **(A)** KEGG pathway enrichment analysis of differentially expressed genes (DEGs) between tissue types (primary tumor vs. adjacent tissue; metastasis vs. primary tumor) for each NK/T cell subset. Dot color indicates cell type, shape indicates tissue comparison, and size reflects gene count. **(B)** Volcano plots showing DEGs in each NK/T cell subpopulation between primary tumors (PT) and Adj_Normal tissues. The x-axis represents cell types, and the y-axis indicates the average log_2_ fold change. Red dots denote significantly upregulated genes, and blue dots denote significantly downregulated genes in primary tumors compared with those in adjacent normal tissues. **(C)** Volcano plots displaying DEGs between metastatic and PT tissues across each NK/T cell subpopulation. Color scheme is consistent with panel **(B, D)** Heatmap showing stage-specific DEGs across NK/T cell subtypes during tumor progression (PM-I to PM-IV). **(E)** Stacked bar plot quantifying up- and downregulated genes for each NK/T cell subtype across tumor stage comparisons. Most transcriptional changes occur between stages I and II, with a predominant trend toward gene downregulation.

Given the observed alterations in NK/T cell composition, we next explored the potential molecular mechanisms underlying these changes. Differential expression analysis followed by KEGG pathway enrichment revealed distinct functional programs between tissues (**Figures 3A-C**; **Supplementary Table 6**). The upregulated pathways in CD4+ TEM cells included ribosome biogenesis, coronavirus infection, human papillomavirus infection, mitogen-activated protein kinase (MAPK) signaling, and PI3K-Akt signaling, implicating these cells in active protein synthesis and immune responses. Notably, CD4+ naïve T cells exhibited pronounced downregulation of the MAPK pathway, highlighting functional suppression in this subset during tumor progression. To further investigate the impact of tumor progression on NK/T cells, we performed pairwise differential expression analysis across primary tumor stages (PM-I to PM-IV). Heatmap visualization revealed widespread stage-dependent transcriptional alterations, particularly between stage I and subsequent stages (**Figure 3D**; **Supplementary Figure 2D**). DEG quantification demonstrated a consistent trend of gene downregulation across NK/T cell subtypes during tumor progression (**Figure 3E**; **Supplementary Table 7**). Most transcriptional changes occurred between stages I and II, suggesting that early-stage tumors mark a critical immunological turning point characterized by the loss of NK/T cell functionality. Collectively, these results reveal profound remodeling of the NK and T cell compartments during GC development and metastasis, with functional impairment emerging as an early event, underscoring the potential windows for immunotherapeutic intervention.

### 3.3 Distinct myeloid cell subtypes exhibit dynamic remodeling and functional suppression during GC progression and metastasis

**Figure 4:**
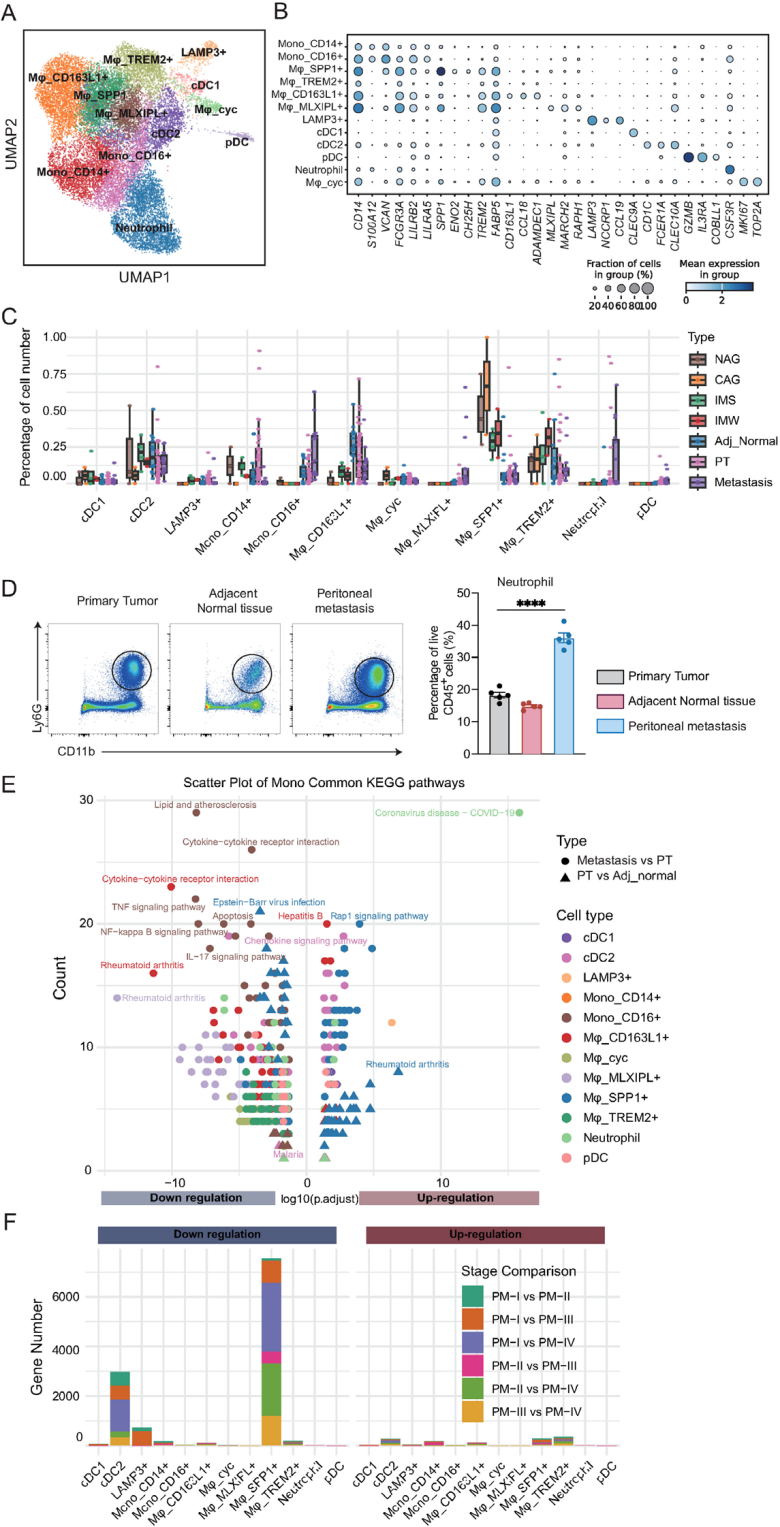
Myeloid cell subtypes exhibit distinct compositional and functional alterations during the development and metastasis of gastric cancer. **(A)** UMAP visualization of 12 transcriptionally distinct myeloid cell subsets, including monocytes, macrophages, dendritic cells, plasmacytoid dendritic cells (pDCs), neutrophils, and proliferating macrophages. **(B)** Dot plot of canonical marker genes defining myeloid cell subsets. Dot size represents the fraction of cells expressing the gene; color intensity reflects average expression. **(C)** Boxplots showing the proportion of each myeloid cell subset across tissue types. CD163L1^+^ macrophages decline in tumors and metastases; MLXIPL^+^ macrophages, pDCs, and TREM2^+^ macrophages increase during disease progression. **(D)** Statistical analysis of neutrophil (CD11b^+^Ly6G^+^) proportions from gastric cancer patients (n = 5/group). One-way ANOVA; ****P < 0.0001. **(E)** KEGG pathway enrichment analysis of differentially expressed genes between tissue types for each myeloid cell subset. Downregulated pathways are enriched for immune-related and inflammatory processes, while upregulated pathways include Rap1 signaling and coronavirus infection. **(F)** Stacked bar plots quantifying up- and downregulated genes for each myeloid subset across primary tumor stages. cDC2 cells exhibit early-stage transcriptional changes; SPP1^+^ macrophages display marked alterations at stage IV, implicating them in metastatic progression.

To further delineate the role of myeloid cells in the TME of GC, we performed a sub-clustering analysis. This analysis identified 12 transcriptionally distinct myeloid cell subsets, including classical and non-classical monocytes, diverse macrophage populations, dendritic cell subtypes, plasmacytoid dendritic cells (pDCs), neutrophils, and proliferating macrophages (**Figure 4A**). These subsets were characterized by canonical marker genes, as demonstrated by dot plot analysis (**Figure 4B**). For instance, CD14 and FCGR3A defined classical and non-classical monocytes, respectively; SPP1, CD163L1, TREM2, and MLXIPL marked distinct macrophage subsets; and CLEC9A, CLEC10A, and IL3RA distinguished between dendritic and pDC populations.

To explore tissue-specific alterations in myeloid cell composition, we quantified the relative abundance of each subset across disease stages (**Figure 4C**; **Supplementary Table 8**). CD163L1+ macrophages were enriched in cancer-adjacent tissues, but declined progressively in primary tumors and metastases, suggesting a potential tumor-suppressive role in this population. In contrast, MLXIPL+ macrophages and pDCs were nearly absent in precancerous tissues, but accumulated during tumor initiation and metastasis. TREM2+ macrophages exhibited a biphasic pattern, increasing during precancerous progression, but decreasing after tumor formation. Unexpectedly, SPP1+ macrophages, previously implicated in tumor promotion, showed relatively stable proportions across disease stages, suggesting a more complex role. Neutrophils were relatively scarce in primary and adjacent normal tissues, but exhibited a marked increase in metastatic lesions, indicating their potential involvement in the metastatic cascade. Consistently, flow cytometry of matched tissues from the same patient showed that neutrophils were the most abundant in the peritoneal metastasis (**Figure 4D**).

To investigate the functional alterations underlying these compositional changes, we performed differential expression and KEGG pathway enrichment analyses comparing primary tumors to adjacent tissues and metastases to primary tumors (**Figure 4E**; **Supplementary Figures 3A–C**; **Supplementary Table 9**). In contrast to NK/T cells, myeloid cells exhibited a predominant downregulation of immune-related pathways, including TNF signaling, NF-kappa B signaling, IL-17 signaling, and cytokine-cytokine receptor interactions, indicating progressive functional suppression. Lipid metabolism and atherosclerosis-related pathways were also downregulated, whereas coronavirus infection and Rap1 signaling were among the few pathways upregulated during tumor progression. SPP1+ macrophages upregulated rheumatoid arthritis-related pathways in metastases, indicating their potential involvement in late-stage disease and metastasis.

Finally, to elucidate the stage-dependent transcriptional dynamics, we performed pairwise differential expression analysis across the primary tumor stages within each myeloid subset (**Figure 4F**; **Supplementary Table 10**). Most subsets exhibited minimal changes in gene expression, indicating their functional stability. However, cDC2 cells displayed a substantial number of DEGs that were predominantly downregulated, with most changes occurring between stage I and later stages, suggesting an early loss of dendritic cell function. SPP1+ macrophages exhibited pronounced transcriptional alterations at stage IV, implying that their functional reprogramming may be linked to tumor metastasis. Collectively, these results reveal profound compositional and functional remodeling of the myeloid compartment during GC progression, characterized by the early impairment of dendritic cells and late-stage activation of tumor-associated macrophages, providing insights into the potential immunoregulatory mechanisms driving disease advancement.

### 3.4 Stromal cell remodeling reveals potential drivers of tumor progression and metastasis

**Figure 5:**
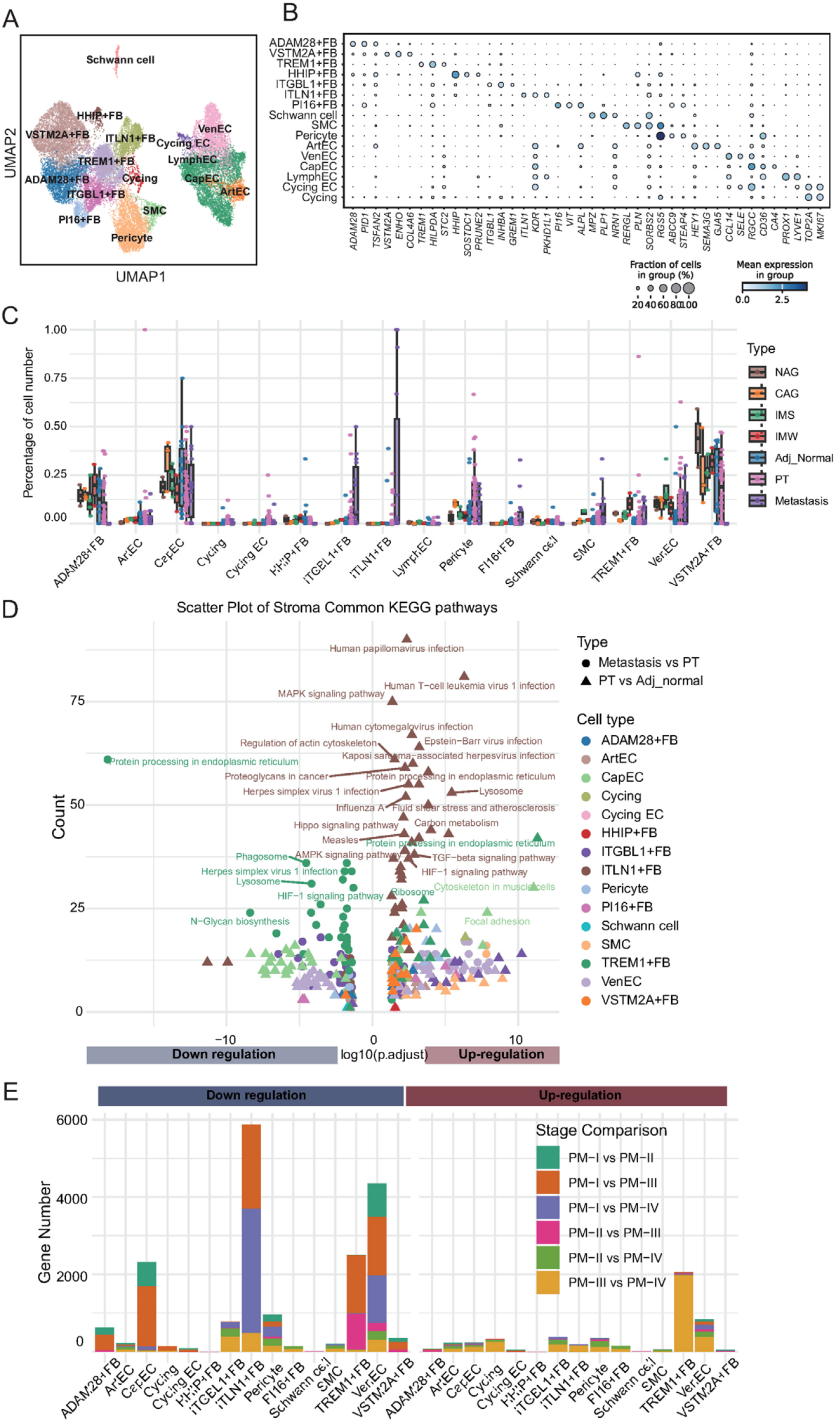
Stromal cell remodeling during gastric cancer progression reveals dynamic changes in abundance and function. **(A)** UMAP visualization of 15 stromal cell subtypes, including fibroblasts, endothelial cells (ArtEC, arterial endothelial cells; VenEC, venous endothelial cells; CapEC, capillary endothelial cells; LymphEC, lymphatic endothelial cells; FB, fibroblast), smooth muscle cells, pericytes, and Schwann cells. **(B)** Dot plot showing expression of representative marker genes for each stromal cell subtype. Dot size reflects the percentage of expressing cells; color intensity indicates average expression. **(C)** Boxplots depicting the relative abundance of stromal cell subtypes across tissue types. ADAM28^+^ fibroblasts decline in tumors; ITGBL1^+^, PI16^+^, TREM1^+^ fibroblasts, and endothelial cells increase with disease progression; ITLN1^+^ fibroblasts emerge in tumors and metastases. **(D)** KEGG pathway enrichment of differentially expressed genes between adjacent normal vs. primary tumors and primary tumors vs. metastases. ITLN1^+^ fibroblasts show upregulation of metastasis-related pathways; TREM1^+^ fibroblasts exhibit downregulation of pathways despite numerical expansion. **(E)** Stacked bar plots summarizing differentially expressed genes between tumor stages for each stromal cell subtype. Stage I vs. later stages account for most transcriptional changes, with ITLN1^+^ fibroblasts and TREM1^+^ fibroblasts displaying distinct stage-dependent expression dynamics.

To investigate the dynamic alterations and potential functions of stromal cells during GC initiation and progression, we performed a detailed sub-cluster analysis of stromal populations. UMAP visualization revealed 15 distinct stromal cell subpopulations, including multiple fibroblast subsets, pericytes, smooth muscle cells (SMCs), ECs, and Schwann cells (**Figure 5A**). The characteristic gene expression profiles of each stromal subpopulation were defined using a dot plot (**Figure 5B**). For example, ADAM28+ fibroblasts expressed high levels of ADAM28, ITGBL1+ fibroblasts expressed ITGBL1, and PI16+ fibroblasts expressed PI16. Additionally, ITLN1+ fibroblasts uniquely expressed ITLN1, TREM1+ fibroblasts expressed TREM1, and venous ECs (VenECs) expressed RGS5.

Next, we analyzed the compositional changes in these subpopulations across different tissue types, including adjacent normal tissues, precancerous lesions, primary tumors, and metastatic tumors (**Figure 5C**; **Supplementary Table 11**). ADAM28+ fibroblasts were enriched in precancerous and adjacent normal tissues but exhibited a marked decrease in primary and metastatic tumors. Given that ADAM28 belongs to the ADAM metalloproteinase family that is involved in ECM homeostasis, the depletion of ADAM28+ fibroblasts likely reflects the disruption of stromal integrity and loss of a protective barrier, thereby facilitating tumor invasion. In contrast, ITGBL1+ fibroblasts, which progressively accumulated from precancerous lesions to primary tumors and metastases, expressed ITGBL1, a protein implicated in promoting ECM deposition, stiffness, and tumor cell migration. This finding suggests that ITGBL1+ fibroblasts actively contribute to the establishment of a pro-tumorigenic microenvironment through matrix remodeling and immune evasion. Similarly, PI16+ fibroblasts, defined by PI16 expression, and SMCs demonstrated a continuous increase in expression during disease progression. PI16 has been linked to fibroblast activation and ECM organization, whereas SMCs are known to generate contractile forces that enhance stromal stiffness and interstitial pressure, both of which promote tumor cell invasion and metastasis. TREM1+ fibroblasts, which progressively increased in tumor and metastatic tissues, expressed TREM1, a receptor classically involved in amplifying inflammatory responses. Their expansion implies a potential role in sustaining chronic inflammation within the tumor stroma, which is a known driver of cancer progression. The proportion of VenECs characterized by RGS5 expression also increased with disease progression. Given the role of RGS5 in vascular remodeling and abnormal angiogenesis, expansion of VenECs may promote tumor neovascularization, facilitating tumor growth and metastatic dissemination. ITLN1+ fibroblasts were nearly absent in precancerous and adjacent normal tissues, but specifically appeared in primary and metastatic tumors. ITLN1 is associated with ECM remodeling and immune regulation. These findings suggest that ITLN1+ fibroblasts represent a metastasis-associated fibroblast subtype that fosters a permissive niche for tumor cell colonization and survival at distant sites.

To explore the potential biological functions of these altered stromal subpopulations, we performed pathway enrichment analysis on DEGs between primary tumors and adjacent normal tissues and between metastatic and primary tumors (**Figure 5D**; **Supplementary Figure 4A–C**; **Supplementary Table 12**). Notably, ITLN1+ fibroblasts in metastatic tissues showed significant enrichment of upregulated pathways, including those related to human papillomavirus infection and lysosomal activity, indicating their involvement in metastatic niche remodeling. In contrast, TREM1+ fibroblasts, despite their increased abundance, exhibited enrichment of downregulated pathways such as protein processing in the endoplasmic reticulum, indicating complex functional reprogramming during metastasis. Finally, we quantified the number of DEGs across tumor stages to further understand stromal evolution during cancer progression (**Figure 5E**; **Supplementary Table 13**). Although ITLN1+ fibroblasts were associated with pathway upregulation in metastasis, they exhibited significant downregulation of gene expression when comparing early- and late-stage primary tumors, particularly between stages I and III/IV. Similar trends were observed for EC subsets, including capillary ECs and VenECs. TREM1+ fibroblasts displayed stage-specific gene expression patterns, with pronounced upregulation of genes in stage III/IV and downregulation in early-stage tumors. Collectively, these results highlight the dynamic remodeling of the stromal compartment during GC progression. Specific fibroblast and endothelial subpopulations, defined by their unique marker gene signatures, undergo distinct compositional and functional changes that may contribute to ECM remodeling, angiogenesis, immune modulation, and establishment of a metastatic microenvironment.

### 3.5 Intercellular communication networks facilitate tumor progression in the GC microenvironment

**Figure 6:**
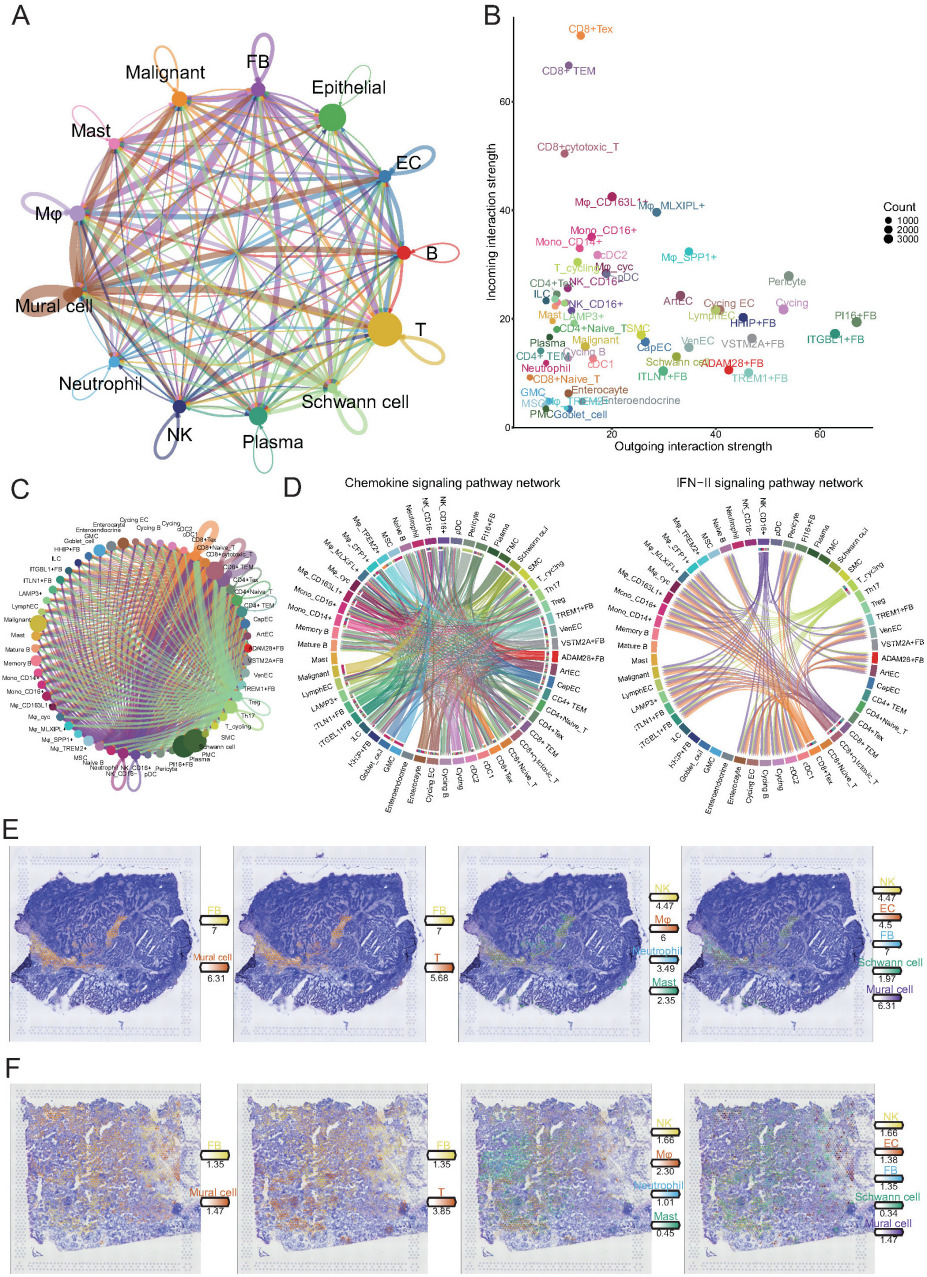
Comprehensive cell-cell interaction analysis reveals key communicative hubs in the gastric cancer microenvironment. **(A)** Circos plot illustrating global intercellular interaction strength among major cell lineages. The width of each connection reflects the strength of interaction. **(B)** Scatter plot showing outgoing (x-axis) and incoming (y-axis) interaction strength of each cell subset. **(C)** Detailed interaction network between NK/T cell subpopulations and all other cell subsets. **(D)** Circos plots depicting cellular communication patterns within the chemokine (left) and IFN-II (right) signaling pathways. In the chemokine network, endothelial cells and fibroblasts mainly act as signal senders, while CD4+ and CD8+ T cells serve as major signal receivers. In the IFN-II network, CD16+ NK cells and CD8+ T cell subsets predominantly act as signal senders targeting myeloid and stromal cells. **(E, F)** Spatial transcriptomics reveal the spatial distribution of major cell lineages in tumor tissue sections. Immune and stromal cells exhibit colocalization within tumor stromal regions, providing spatial evidence for potential direct cellular interactions.

To investigate how interactions among diverse cell populations shape the GC microenvironment and promote tumor progression, we performed comprehensive cell-cell interaction analyses across all major cellular compartments. At the global level, we constructed an intercellular communication network among major cell lineages (**Figure 6A**). This analysis revealed that fibroblasts and mural cells exhibit the strongest overall interaction strength with other cell types, highlighting their central role as organizational hubs within the TME. Given the known contributions of fibroblasts to ECM remodeling, immunomodulation, and angiogenesis, their prominent interactions likely facilitate the establishment of a tumor-permissive niche that supports cancer cell survival, immune evasion, and metastatic dissemination.

To further delineate the directionality and functional implications of these interactions, we quantified the strengths of the outgoing (signal-sending) and incoming (signal-receiving) interactions for each cellular subpopulation (**Figure 6B**). CD8+ T cell subsets, including exhausted T cells (Tex), effector memory T cells (TEM), and cytotoxic T cells, exhibited markedly higher incoming interaction strengths, suggesting that these key antitumor effector populations are subject to extensive external modulation within the TME. In contrast, fibroblast subpopulations, particularly ITGBL1+, PI16+, and VSTM2A+ fibroblasts, displayed dominant outgoing interaction strengths, implicating them as major signaling sources that actively influence the behavior of neighboring cells. These findings suggest that tumor-associated fibroblasts not only provide structural support but also act as potent regulators of immune cell function, potentially contributing to T cell dysfunction and immune suppression.

Given the critical role of T cells in tumor control, we systematically examined their interactions with other cell types (**Figure 6C**). Notably, CD8+ Tex, TEM, and cytotoxic T cells exhibited the most extensive and robust crosstalk with diverse cell populations, reinforcing the concept that these effector populations, despite their dysfunctional state, remain the central nodes of cellular communication within the tumor. The pervasive interactions between fibroblasts and exhausted T cells imply a feed-forward loop, in which stromal-derived signals further impair T cell function, ultimately promoting immune escape and tumor progression.

To elucidate specific signaling pathways involved in these interactions, we focused on chemokine and type II interferon (IFN-γ) signaling axes, both of which regulate immune cell recruitment and activation (**Figure 6D**). Within the chemokine network, fibroblasts and ECs primarily acted as signal senders, whereas CD4+ and CD8+ T cells served as major signal recipients. This result suggests that stromal cells orchestrate immune cell positioning within the tumor, which may favor the formation of immunosuppressive niches. In contrast, IFN-γ signaling was predominantly initiated by CD16+ NK cells, CD8+ TEM, cytotoxic T cells, and Tex cells, targeting various myeloid and stromal populations. Although IFN-γ is traditionally associated with antitumor immunity, its dysregulated production within an immune-suppressive environment may paradoxically contribute to chronic inflammation and stromal reprogramming, thereby supporting tumor progression.

Finally, to validate these predicted intercellular interactions at the spatial level, we integrated the spatial transcriptomic data (**Figures 6E, F**). Immune and stromal cells, particularly fibroblasts and mural cells, exhibited pronounced spatial colocalization within tumor stromal regions. This spatial proximity provides a structural basis for direct cellular crosstalk, facilitating the transmission of pro-tumorigenic signals. Together, these findings reveal a complex and coordinated intercellular communication network in the gastric TME, in which tumor-associated fibroblasts emerge as key regulators driving immune dysfunction and stromal remodeling. This network promotes immune evasion, tumor progression, and metastasis via extensive crosstalk with immune cells, particularly CD8+ T cells.

### 3.6 Identification of stage-associated gene modules by WGCNA

To identify the gene modules associated with tumor progression and immune cell alterations, we performed WGCNA based on TCGA cohort transcriptomic data. Considering our previous findings that the DEGs of NK/T, myeloid, and stromal cells vary significantly across tumor stages, we first applied ssGSEA to calculate the DE scores for these three cell types across samples.

**Figure 7:**
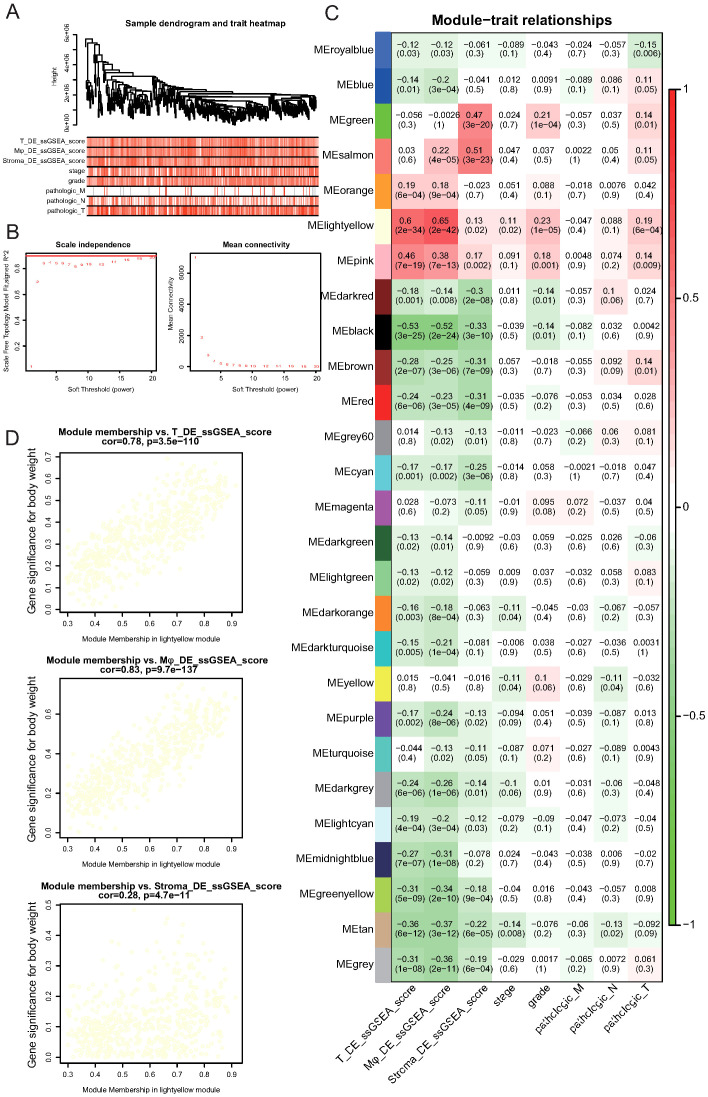
Identification of gene modules associated with immune cell alterations and tumor progression by Weighted Gene Co-expression Network Analysis (WGCNA). **(A)** Sample clustering dendrogram and corresponding trait heatmap. ssGSEA scores for NK/T cells (T_DE_ssGSEA_score), myeloid cells (Mφ_DE_ssGSEA_score), stromal cells (Stroma_DE_ssGSEA_score), and clinical features, including tumor stage, grade, and pathological TNM classifications, are shown. **(B)** Determination of soft-thresholding power for WGCNA. The left panel shows the scale-free topology fit index (R²) versus soft-thresholding power. A power of 3 achieves an R² above 0.9 (red line). The right panel shows mean connectivity across powers. **(C)** Heatmap of module–trait correlations. Each cell displays the correlation coefficient and corresponding p-value between the module eigengene and the indicated trait. The lightyellow module shows the strongest positive association with NK/T cell and myeloid cell DE scores. **(D)** Scatter plots showing correlations between gene significance for NK/T cell DE scores, myeloid cell DE scores, or stromal cell DE scores and module membership in the lightyellow module. Strong positive correlations were observed for NK/T and myeloid cell DE scores, indicating the biological relevance of this module.

A hierarchical clustering dendrogram combined with a trait heatmap revealed clear clustering patterns among samples, with DE scores and clinical traits such as tumor stage and pathological features showing distinct distributions (**Figure 7A**). Next, to ensure the construction of a scale-free co-expression network, we selected a soft-thresholding power of 3, which satisfied the scale-free topology criterion with R² exceeding 0.9 (**Figure 7B**).

Module-trait correlation analysis identified multiple modules that were significantly associated with immune cell DE scores and clinical features (**Figure 7C**; **Supplementary Table 14**). Notably, the “lightyellow” module demonstrated the strongest positive correlation with NK/T cell DE scores (r = 0.60, p = 2.4e-34) and myeloid cell DE scores (r = 0.46, p = 7e-19) and showed associations with tumor stage and pathological indicators (**Figure 7C**). Further assessment revealed a strong positive correlation between gene significance for NK/T cell DE scores and module membership within the lightyellow module (r = 0.78, p = 3.5e-110; **Figure 7D**). Similarly, the gene significance for myeloid cell DE scores exhibited an even stronger correlation with module membership in the lightyellow module (r = 0.83, p = 9.7e-137). In contrast, although stromal cell DE scores were also correlated with the lightyellow module, the association was notably weaker (r = 0.28, p = 4.7e-11). Collectively, these results indicate that the lightyellow module, which contains 657 genes, is closely associated with tumor progression and immune cell alterations, particularly those involving NK/T and myeloid cells, suggesting that this gene set plays a crucial role in shaping the TME during GC progression.

### 3.7 Deep learning-based prognostic model for GC

**Figure 8:**
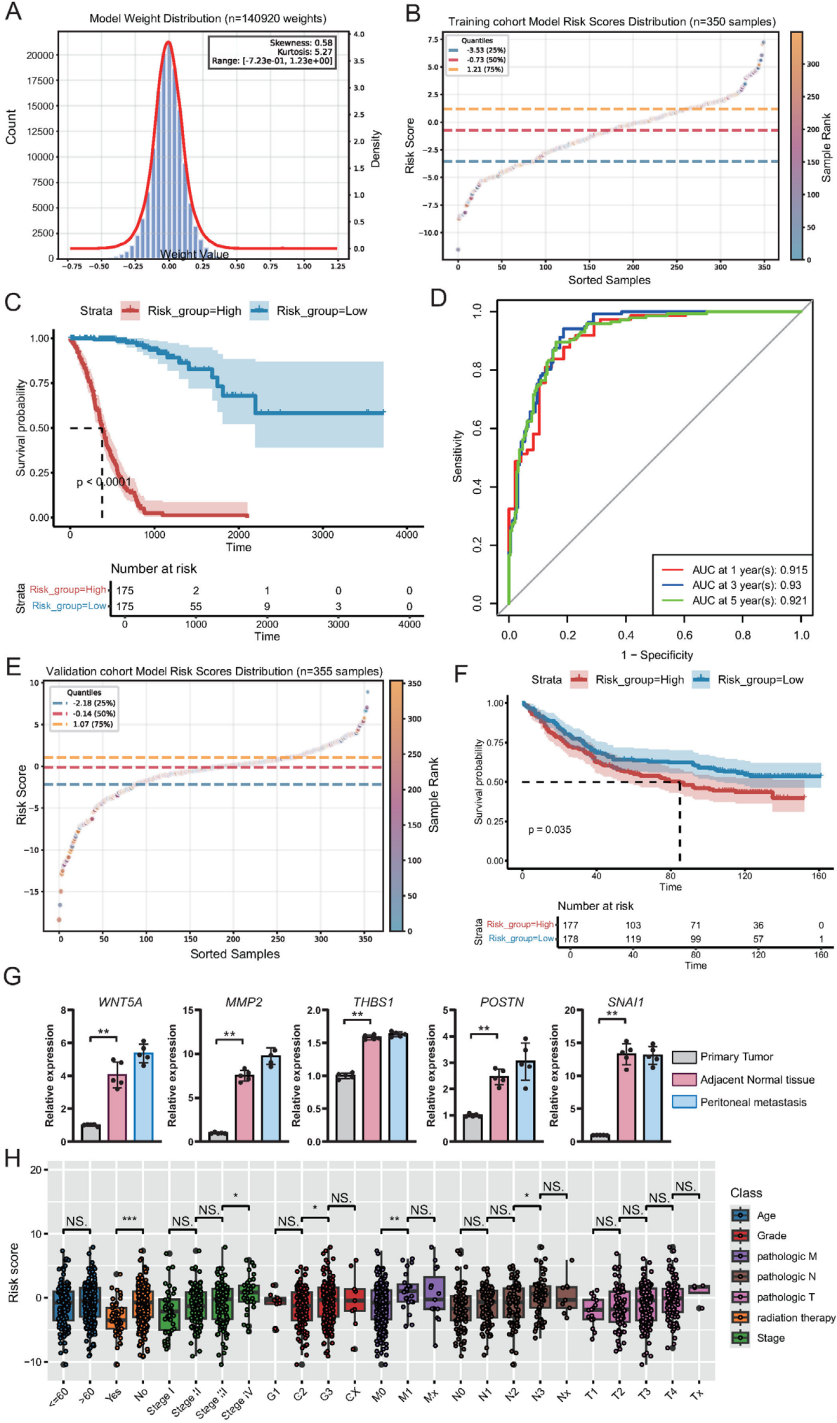
Construction and validation of a deep learning-based prognostic model for gastric cancer. **(A)** Histogram and density curve illustrating the distribution of model weights (n = 140, 920), showing near-normal distribution with minor skewness and kurtosis. **(B)** Scatter plot showing the distribution of risk scores across the training cohort (n = 350), with quartile thresholds indicated by dashed lines. **(C)** Kaplan-Meier survival curves for high- and low-risk groups in the training cohort, demonstrating significantly poorer survival in the high-risk group (p < 0.0001). **(D)** Time-dependent ROC curves for the training cohort showing excellent predictive accuracy for 1-, 3-, and 5-year overall survival. **(E)** Scatter plot showing the risk score distribution in the independent validation cohort (n = 355). **(F)** Kaplan-Meier survival analysis in the validation cohort confirming significantly worse survival in the high-risk group (p = 0.035). **(G)** qPCR demonstrated the differential expression of the top five genes of the deep learning model in adjacent, primary tumor, and peritoneal metastatic tissues. **(H)** Boxplots displaying the distribution of risk scores across different clinical subgroups within the training cohort. Statistically significant differences were observed in relation to radiation therapy status, tumor stage, histological grade, and lymph node metastasis, supporting the clinical relevance of the model-derived risk score.

To establish a prognostic model for GC based on previously identified tumor stage-associated gene modules, we employed a deep learning approach using the TCGA GC cohort as the training dataset. The model weight distribution followed an approximately normal distribution, as shown in the histogram, indicating the robustness of model parameter initialization (**Figure 8A**). The distribution of individual risk scores calculated using the model revealed a continuous and widespread distribution across patients in the training cohort (**Figure 8B**; **Supplementary Table 15**). Kaplan-Meier survival analysis demonstrated that patients classified into the high-risk group exhibited significantly worse overall survival than those in the low-risk group (p < 0.0001; **Figure 8C**). Furthermore, time-dependent ROC curve analysis showed excellent predictive performance, with the area under the curve reaching 0.915, 0.930, and 0.921 for 1-, 3-, and 5-year survival, respectively (**Figure 8D**).

To validate the predictive power of this model, we applied it to an independent external validation cohort. Similar to that in the training set, the risk score distribution in the validation cohort varied widely (**Figure 8E**; **Supplementary Table 16**). High-risk patients in the validation cohort consistently had significantly worse survival outcomes than low-risk patients (p = 0.035; **Figure 8F**), confirming the robust prognostic capability of the model. Subsequently, we examined the expression differences of the top five genes constructed in adjacent tissues, primary GC tissues, and peritoneal metastatic tissues. The results showed that the expression of these genes was significantly different in different tissues, specifically manifesting as the lowest expression in the adjacent tissues and an upward trend in primary GC and peritoneal metastasis tissues (**Figure 8G**).

Furthermore, we explored the association between risk scores and clinical features in the training cohort. Boxplot analysis revealed that the risk scores were significantly higher in patients who did not receive radiation therapy, as well as in patients with a more advanced tumor stage (stage III vs. stage II; stage IV vs. stage III), higher histological grade (G3 vs. G2), and more severe lymph node involvement (N2 vs. N1) (**Figure 8H**). These results indicate that the model-derived risk score not only reflects patients’ prognostic outcomes but also correlates with classical clinicopathological indicators of disease severity.

## 4 Discussion

In this study, we comprehensively delineated the cellular ecosystem and intercellular interactions underlying GC progression and metastasis using scRNA-seq, spatial transcriptomics, bulk transcriptomic analysis, and deep learning-based prognostic modeling. Our results provide novel insights into the dynamic remodeling of the immune and stromal compartments, highlight specific cell subpopulations and communication patterns that drive tumor progression, and offer a clinically applicable prognostic model with strong predictive power.

First, by constructing a high-resolution single-cell atlas of GC and related tissue states, we revealed profound heterogeneity within the TME, which is consistent with previous studies on GC and other solid tumors (27). Notably, the progressive accumulation of dysfunctional CD8+ T cells, immunosuppressive myeloid populations, and pro-tumorigenic fibroblast subsets underscores the coordinated remodeling of the TME to facilitate immune evasion and tumor progression (28, 29). Our trajectory analysis further demonstrated impaired T-cell differentiation and functional exhaustion during tumor development, echoing observations in lung and colorectal cancers (8, 30). We identified specific stromal subpopulations, including ITGBL1+ and PI16+ fibroblasts, TREM1+ fibroblasts, and ITLN1+ fibroblasts, which displayed dynamic alterations and potential pro-tumorigenic functions based on characteristic gene expression patterns and pathway enrichment. The association of ITGBL1 with ECM remodeling and immune evasion has been reported in ovarian and colorectal cancers (31, 32), whereas PI16 has been implicated in fibrotic diseases and the activation of tumor-associated fibroblasts (33). Moreover, our identification of ITLN1+ fibroblasts as a metastasis-associated population extends recent findings linking ITLN1 to matrix remodeling and tumor dissemination in colorectal cancers (34).

Our cell-cell interaction analysis revealed extensive crosstalk between stromal and immune cells, with fibroblasts acting as the dominant signal source and exhausted CD8+ T cells as major signal recipients. This observation is consistent with reports that cancer-associated fibroblasts actively modulate T cell dysfunction and exclusion through cytokine and chemokine signaling (35, 36). Notably, IFN-γ signaling, typically regarded as anti-tumorigenic, was predominantly initiated by dysfunctional T cells in our cohort, potentially contributing to chronic inflammation and further stromal reprogramming, a mechanism increasingly recognized in tumor biology (37, 38).

Integration of our single-cell and spatial transcriptomic data further confirmed the spatial organization of immune and stromal cells within tumor tissues, providing structural evidence for functional crosstalk. Similar spatially resolved studies have emphasized the importance of immune-stromal niches in regulating tumor progression and the response to therapy (11, 13). To translate these biological insights into clinical utility, we constructed a deep learning-based prognostic model using stage-associated gene modules derived from bulk transcriptomic and co-expression network analyses. Our model demonstrated excellent performance in the training and independent validation cohorts, outperforming conventional clinical parameters. Previous studies have highlighted the potential of machine learning and multi-omics integration for prognostic prediction in lung adenocarcinoma (39, 40). However, our model is distinguished by its direct biological grounding in single-cell and spatial transcriptomic alterations, providing predictive value and mechanistic interpretability.

Despite these strengths, several limitations of this study must be acknowledged. First, although our study used a large and diverse sample cohort, future validation in larger prospective clinical trials is warranted. Second, the functional validation of specific stromal and immune cell subpopulations, particularly ITLN1+ fibroblasts, is necessary to confirm their role in metastasis. Finally, the incorporation of additional multi-omics data, such as proteomics or epigenomics, could further refine the mechanistic understanding of TME remodeling in GC.

In conclusion, our integrated single-cell, spatial, and computational analysis provides a comprehensive framework for understanding TME remodeling during GC progression. The identification of key cellular subpopulations and intercellular interactions, coupled with the development of a clinically applicable prognostic model, offers new opportunities for biomarker discovery and therapeutic targeting in GC.

## Data Availability

The original contributions presented in the study are included in the article/**Supplementary Material**. Further inquiries can be directed to the corresponding authors.
