# Supplementary material for: Integrative multi-omics analysis of gastric cancer evolution from precancerous lesions to metastasis identifies a deep learning-based prognostic model
Source: Front Immunol. 2025 Oct 31;16:1680517. doi: 10.3389/fimmu.2025.1680517 (PMC12615467; doi:10.3389/fimmu.2025.1680517)
Supplement: Supplementary file 2 [file DataSheet1.docx]

Supplementary Material

# Supplementary Figures


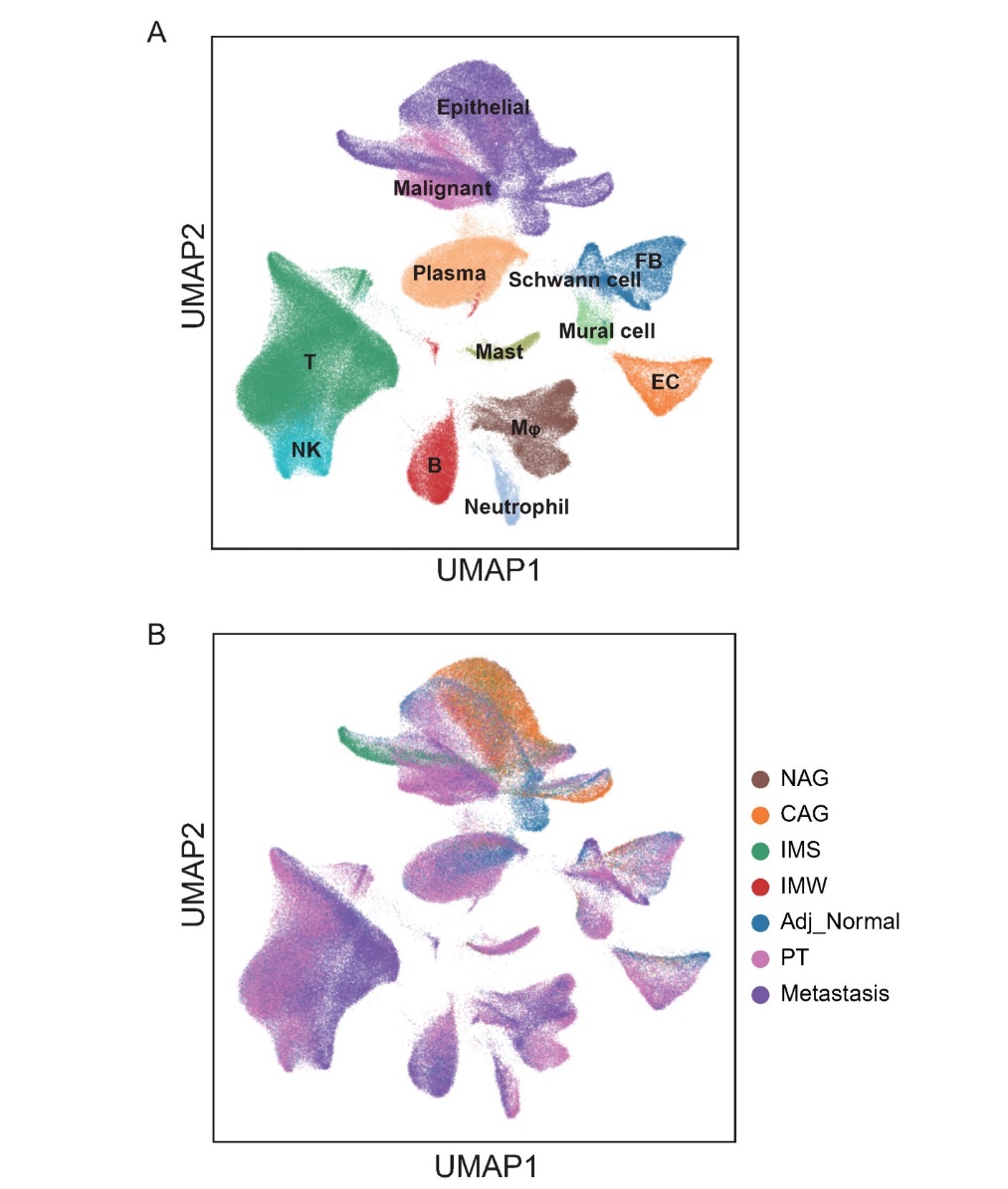


**Supplementary Figure 1.** Broad cell type annotation and tissue origin distribution of single-cell transcriptomic data. (**A**) UMAP visualization of all single-cell transcriptomic data colored by broad cell type annotations. Major cell populations including epithelial cells, malignant cells, fibroblasts (FB), endothelial cells (EC), mural cells, Schwann cells, mast cells, macrophages (Mφ), neutrophils, B cells, T cells, natural killer (NK) cells, and plasma cells are distinctly clustered based on transcriptional profiles. (**B**) UMAP plot showing the distribution of cells derived from seven different tissue types, including non-atrophic gastritis (NAG), chronic atrophic gastritis (CAG), intestinal metaplasia with severe dysplasia (IMS), intestinal metaplasia without severe dysplasia (IMW), adjacent normal tissue (Adj_Normal), primary gastric tumor tissue (PT), and metastatic tissue (Metastasis). Cells from different tissue sources show distinct yet partially overlapping distributions, reflecting progressive changes in cellular composition and transcriptional states during gastric cancer development and metastasis.


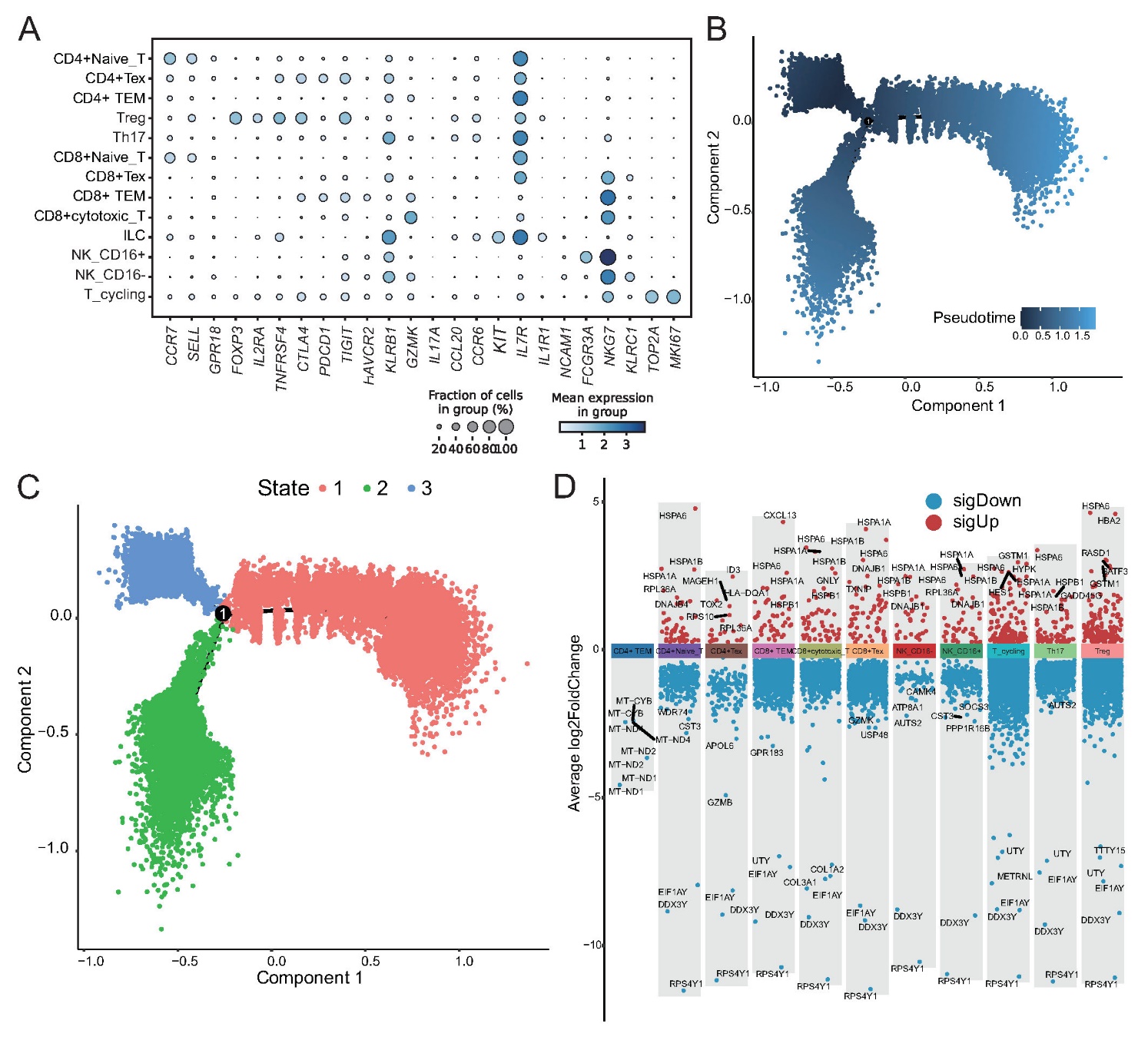


**Supplementary Figure 2.** Characterization and differential expression analysis of NK/T cell subpopulations. (**A**) Dot plot showing the expression levels and percentage of expressing cells for representative marker genes across identified NK/T cell subpopulations, including CD4+ T cell subsets, CD8+ T cell subsets, NK cell subsets, and innate lymphoid cells (ILCs). Dot size represents the percentage of cells expressing the gene within the cluster, and color intensity represents the average expression level. (**B**) Pseudotime trajectory of T cells inferred by Monocle2, illustrating the dynamic differentiation process. Each dot represents a single cell, colored by pseudotime value. (**C**) State classification along the pseudotime trajectory, with cells assigned to three distinct differentiation states, represented by different colors (red: state 1; green: state 2; blue: state 3). (**D**) Volcano plots depicting DEGs among different clinical stages within primary tumor samples across NK/T cell subpopulations. Color scheme is consistent with panel D.


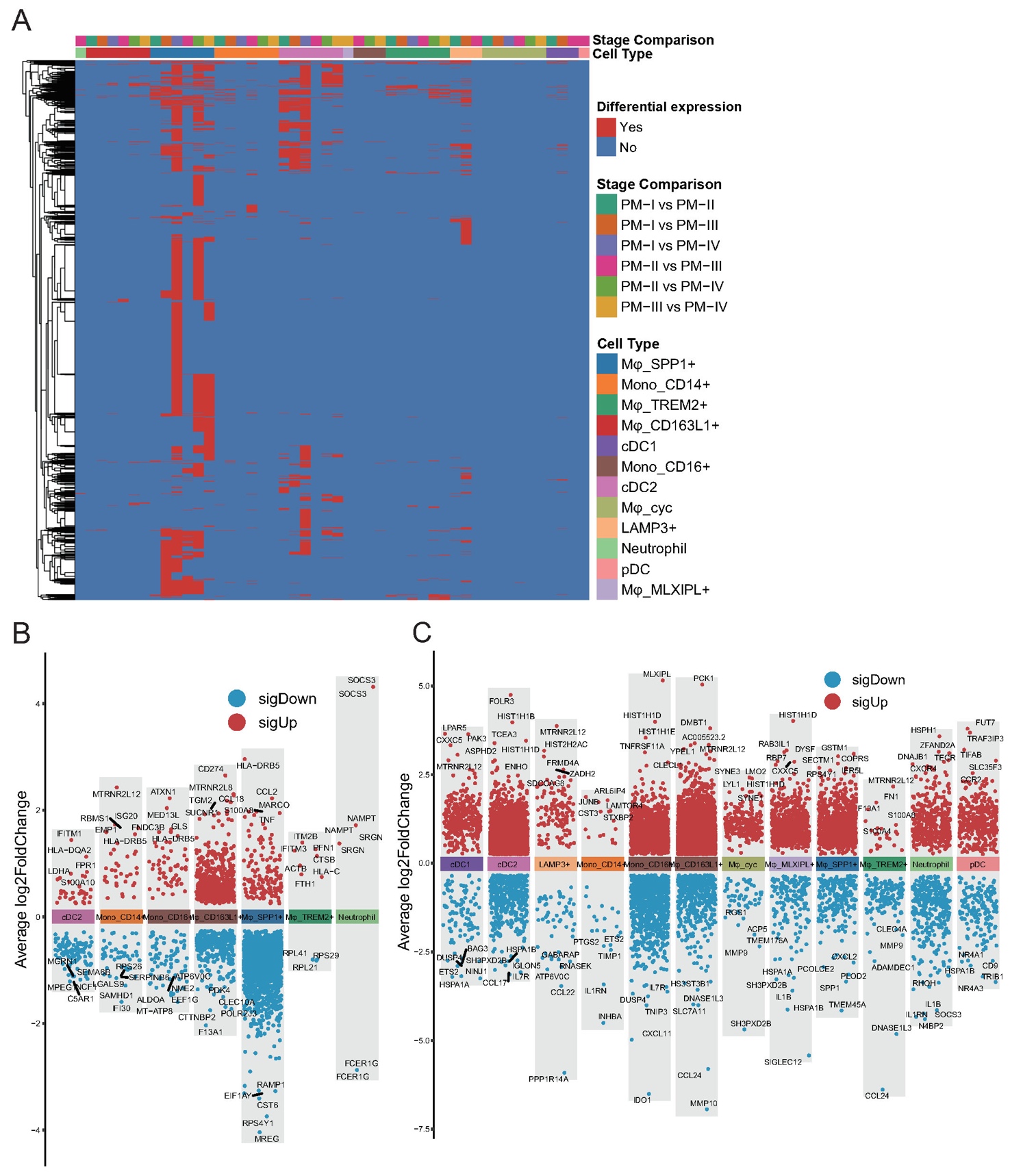


**Supplementary Figure 3.** Differential expression analysis of myeloid cell subsets across tumor progression and tissue comparisons. (**A**) Heatmap showing the distribution of DEGs across various myeloid cell types and tumor stages. The top annotations indicate the stage comparison groups, and the cell type annotations are shown on the right. Red represents significant differential expression, and blue indicates no significant difference. (**B**) Volcano-style scatter plot illustrating the gene expression differences between PT and Adj_Normal within each myeloid cell subset. The x-axis represents cell type, and the y-axis shows the average log2 fold change of gene expression. Red dots represent significantly upregulated genes, and blue dots represent significantly downregulated genes. (**C**) Volcano-style scatter plot showing the gene expression differences between metastatic lesions and primary tumors within each myeloid cell subset. The x-axis represents cell type, and the y-axis indicates the average log2 fold change. Red dots represent significantly upregulated genes, and blue dots represent significantly downregulated gene.


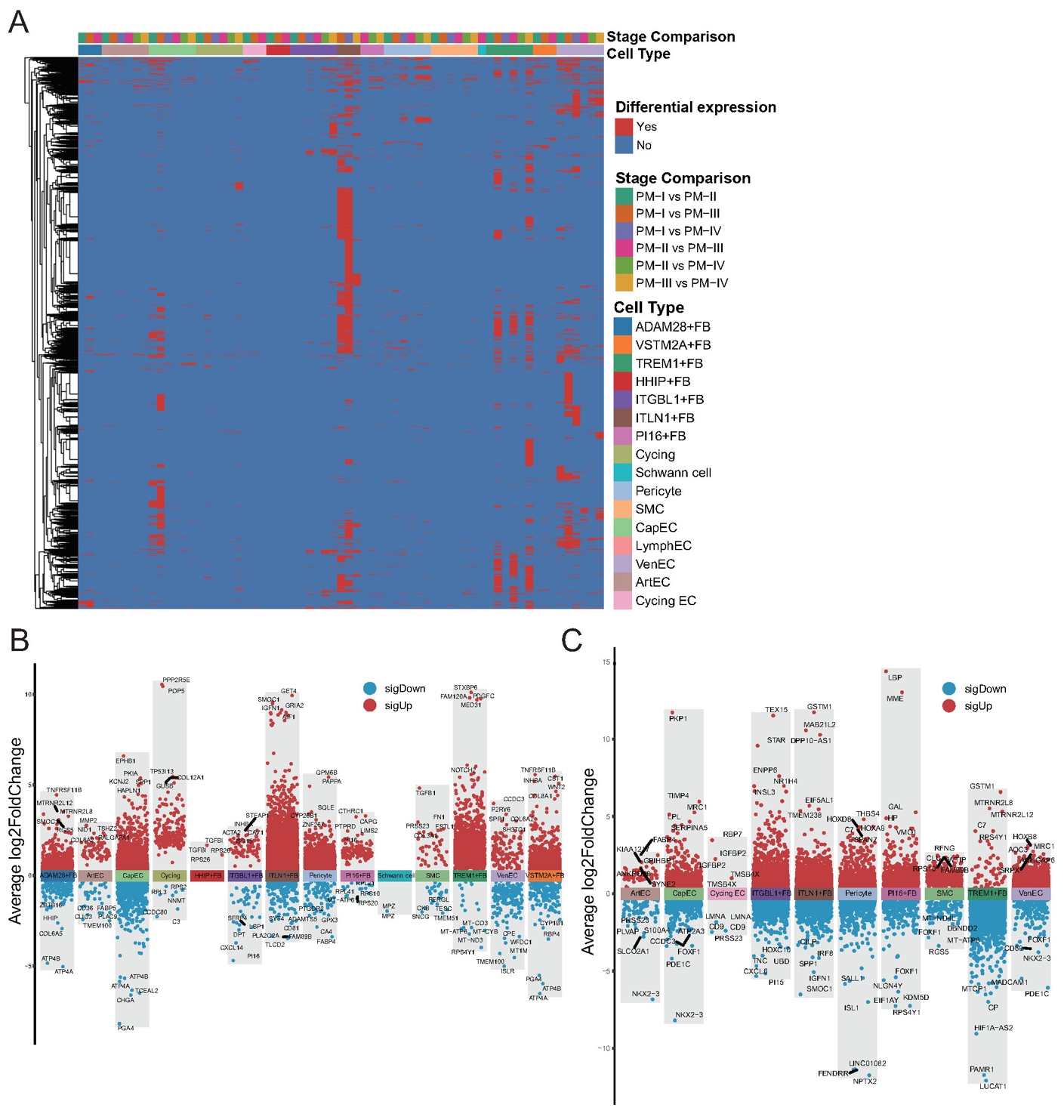


**Supplementary Figure 4.** Differential gene expression analysis of stromal cell subpopulations across different stages and tissue types. (**A**) Heatmap showing DEGs across stromal cell subtypes at various tumor stages. Columns represent different comparisons between tumor stages (PM-I to PM-IV), and rows correspond to individual DEGs. Red indicates significant differential expression, while blue indicates no significant difference. Cell type annotations are shown on the right. (**B**) Volcano plot showing DEGs between primary tumor and adjacent normal tissue for each stromal cell subtype. The x-axis represents cell types, and the y-axis represents the average log2 fold change. Red dots indicate significantly upregulated genes, and blue dots indicate significantly downregulated genes in primary tumors compared to adjacent normal tissue. (**C**) Volcano plot showing DEGs between metastatic and primary tumor samples for each stromal cell subtype. The x-axis represents cell types, and the y-axis represents the average log2 fold change. Red dots indicate significantly upregulated genes, and blue dots indicate significantly downregulated genes in metastatic tumors compared to primary tumors.
